# Substitution of perioperative albumin deficiency disorders (SuperAdd) in adults undergoing vascular, abdominal, trauma, or orthopedic surgery: protocol for a randomized controlled trial

**DOI:** 10.1186/s13063-020-04626-2

**Published:** 2020-08-18

**Authors:** Stefan J. Schaller, Kristina Fuest, Bernhard Ulm, Sebastian Schmid, Catherina Bubb, Rüdiger von Eisenhart-Rothe, Helmut Friess, Chlodwig Kirchhoff, Thomas Stadlbauer, Peter Luppa, Manfred Blobner, Bettina Jungwirth

**Affiliations:** 1 Department of Anesthesiology and Intensive Care, Technical University of Munich, Klinikum rechts der Isar, Ismaninger Straße 22, 81675 Munich, Germany; 2 Department of Anesthesiology and Operative Intensive Care Medicine, Charité – Universitätsmedizin Berlin, corporate member of Freie Universität Berlin, Humboldt-Universität zu Berlin, and Berlin Institute of Health, 10117 Berlin, Germany; 3 Department of Anesthesiology, Ulm University, Albert-Einstein-Allee 23, 89081 Ulm, Germany; 4 Department of Orthopedics, Technical University of Munich, Klinikum rechts der Isar, Ismaninger Straße 22, 81675 Munich, Germany; 5 Department of Surgery, Technical University of Munich, Klinikum rechts der Isar, Ismaninger Straße 22, 81675 Munich, Germany; 6 Department of Traumatology, Technical University of Munich, Klinikum rechts der Isar, Ismaninger Straße 22, 81675 Munich, Germany; 7 Department of Vascular Surgery, Technical University of Munich, Klinikum rechts der Isar, Ismaninger Straße 22, 81675 Munich, Germany; 8 Institute of Clinical Chemistry and Pathobiochemistry, Technical University of Munich, Klinikum rechts der Isar, Ismaninger Straße 22, 81675 Munich, Germany

**Keywords:** Serum albumin, Perioperative care, Postoperative care, Hypalbuminemia, Postoperative complications

## Abstract

**Background:**

Hypalbuminemia is associated with numerous postoperative complications, so a perioperative albumin substitution is often considered. The objective of SuperAdd is to investigate whether substitution of human albumin, aiming to maintain a serum concentration > 30 g/l, can reduce postoperative complications in normovolemic surgical patients in comparison with standard care.

**Methods/design:**

SuperAdd is a single-center, prospective, randomized, outcome-assessor blinded, patient blinded controlled trial. The primary outcome is the frequency of postoperative complications identified using the Postoperative Morbidity Survey graded ≥ 2 according to the Clavien-Dindo Score. Adult patients at risk to develop hypalbuminemia, i.e., ASA III or IV or high-risk surgery, are recruited after written informed consent was obtained. The albumin concentration is assessed before the induction of anesthesia and every 3 h until admission to the postanesthesia care unit. If albumin concentrations drop below 30 g/l, patients are randomly allocated to the control or the treatment group. The study intervention is a goal-directed human albumin substitution aimed at a concentration > 30 g/l during surgery and postanesthesia care unit stay. The patients in the control group are treated according to standard clinical care. Postoperative visits are to be performed on days 1, 3, 5, 8, and 15, as well as by telephone 6 months after surgery.

**Discussion:**

SuperAdd is the first clinical trial in a surgical population investigating the effect of a goal-directed albumin substitution aiming at a serum level > 30 g/l. The nonrestrictive selection of patients guarantees that the patients without albumin screening will most likely not develop hypalbuminemia, thus ensuring generalizability of the study results.

**Trial registration:**

EudraCT 2016-001313-24. Registered on 5 September 2016. Clinical Trials NCT03167645. Registered on 18 October 2016 and has the Universal Trial Number (UTN) U1111-1181-2625.

## Background

Postoperative complications contribute significantly to morbidity and mortality. A recent analysis suggests that postoperative mortality is the third leading cause of death, with at least 4.2 million people worldwide dying within 30 days of surgery each year [1]. Accordingly, perioperative research often focuses on prevention and early recognition of postoperative complications. Hypalbuminemia warrants special consideration due to its association with numerous postoperative complications [2–4]. Hypalbuminemia can preexist as a result of a poor preoperative nutritional status, critical or chronic illness, an insufficient hepatic synthesis [5], or can be acquired over the perioperative course due to bleeding or capillary leakage [6].

As an integral component for homeostasis, perioperative hypalbuminemia may promote postoperative complications. Albumin is the major binding and transporting protein of hormones, coagulation factors [7], and drugs. Furthermore, albumin is a key determinant of oncotic pressure in the vascular system [8], which is a prerequisite for organ perfusion [9]. Therefore, it seems reasonable to consider perioperative albumin substitution.

Aside from studies in intensive care patients [10–12], there are only retrospective [13] or small prospective studies in the perioperative setting, with conflicting results [14, 15] and limited significance. These studies were heterogeneous, analyzing different indications, such as treatment of hypovolemia or hypalbuminemia, different regimes of substitution, and different outcome measures. Accordingly, the clinical question as to whether perioperative albumin deficits should be substituted in the perioperative setting cannot be answered based on the published knowledge. We hypothesize that a goal-directed albumin substitution is likely to be beneficial in surgical patients with an acute loss of albumin.

The aim of the SuperAdd trial is to investigate whether substitution of human albumin, aiming to maintain a serum concentration > 30 g/l, is able to reduce postoperative complications in normovolemic surgical patients as compared to standard care.

## Methods

### Study setting

**Fig. 1:**
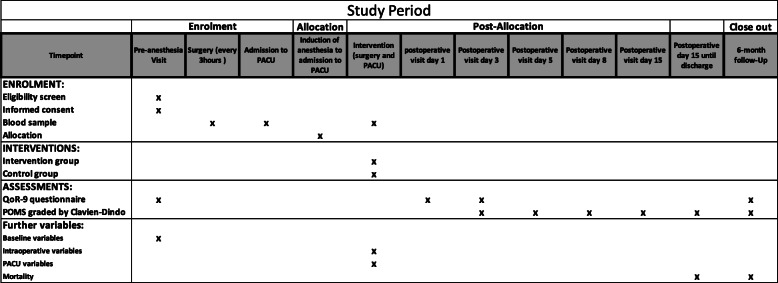
Schedule of enrolment, interventions, and assessments according to the SPIRIT guidelines [16]. PACU, postanesthesia care unit. The Postoperative Morbidity Survey (POMS) screens nine domains (pulmonary, infectious, cardiovascular, neurological, renal, gastrointestinal, wound, pain, and hematological morbidities) [17]. The severity of the complication is graded according to the Clavien-Dindo Score (Table 1) [18]

The SuperAdd trial is a single-center, prospective, randomized, outcome-assessor blinded, controlled trial performed in a university hospital (Technical University of Munich, Klinikum rechts der Isar, Germany). The SuperAdd workflow is provided in Fig. 1 and the SPIRIT Checklist as Additional file 1.

### Eligibility criteria

Patients scheduled for surgery in the departments of vascular, abdominal, trauma, or orthopedic surgery are recruited during the preoperative anesthesia visit. Inclusion criteria include: (1) ≥ 18 years old, (2) written informed consent, and (3) being at risk for the development of hypalbuminemia, which was assumed for either (3a) American Society of Anesthesiologists status III or IV or (3b) high-risk surgery such as esophagectomy, liver surgery, pancreatic surgery, open aortic surgery, open leg revascularization, thrombectomy, limb amputation, or revision of knee and hip arthroplasty.

Exclusion criteria are (1) emergency surgery, (2) severe liver cirrhosis (Child-Pugh score C), (3) terminal kidney failure requiring dialysis, (4) patients previously randomized to this trial or participation in another clinical trial in the past three months, in accordance with German medication laws, (5) patients who have a legal guardian for medical decisions, (6) patients with a history of allergy to albumin or auxiliary substances, (7) pregnant women, (8) breastfeeding women, and (9) patients with a body mass index > 35 kg/m^2^.

### Interventions

The study intervention is a goal-directed albumin substitution with boluses of 20 g human albumin given in 100-ml bottles (Human Albumin 20% Behring™, CSL Behring, Marburg, Germany) to reach albumin serum concentrations ≥ 30 g/l throughout surgery and during the postoperative anesthesia care unit (PACU) stay. Albumin solutions will be infused in whole bottles. The number of bottles is related to the difference between the actual and target albumin concentration and the patient’s body weight:

$$ \mathrm{number}\ \mathrm{of}\ \mathrm{albumin}\ \mathrm{bottles}=\left(\mathbf{30}\ \mathbf{g}/\mathbf{l}-\mathrm{albumin}\ \mathrm{concentration}\right)\times \mathrm{bodyweight}\times 4\ \mathrm{l}/{\mathrm{kg}}^2 $$

The effect is controlled and if necessary escalated.

According to standard clinical care, patients of the control group will be not treated with albumin. If hemodynamic instability persists despite normovolemia, exceptions will be allowed, but the target will remain a serum albumin concentration of 20 g/l. The maximum allowed number of albumin bottles is accordingly:

$$ \mathrm{number}\ \mathrm{of}\ \mathrm{albumin}\ \mathrm{bottles}=\left(\mathbf{20}\ \mathbf{g}/\mathbf{l}-\mathrm{albumin}\ \mathrm{concentration}\right)\times \mathrm{bodyweight}\times 4\ \mathrm{l}/{\mathrm{kg}}^2. $$

### Blinding (masking)

The intervention is performed by the unblinded anesthesiologists in the operation room and PACU. Patients remain blinded, and the outcome is assessed by blinded physicians of the corresponding surgical department, assisted by blinded study staff. Neither the albumin concentration nor the infusion is documented on the laboratory report, the anesthesia report, or the PACU report. Statistical analyses as well as the interim analyses are undertaken independently by statisticians blinded to the treatment group allocation.

### Anesthesia and fluid resuscitation

All patients receive anesthesia according to the hospital’s standard procedures for patients with ASA 3 or 4 status or high-risk surgery [19]. In particular, fluid resuscitation guarantees normovolemia using balanced crystalloids and gelatine solutions, in accordance with the German Guidelines for intraoperative fluid substitution [20]. Major bleedings and coagulation disorders are treated according to the German Guidelines for therapy with blood compounds and plasma derivatives [21]. Exceptions include the treatment with fresh frozen plasma and albumin preparations. While albumin is only allowed according to the study protocol, the infusion of fresh frozen plasma is prohibited due to its albumin content, and its usage represents a major protocol violation.

### Outcomes measures

The primary outcome measure is the occurrence and severity of postoperative complications, identified using the Postoperative Morbidity Survey (POMS) assessed on postoperative days 3, 5, 8, and 15 and at hospital discharge, and graded according to the Clavien-Dindo Score. In brief, the POMS counts the number of domains, in which a complication leading to a prolongation of hospitalization occurs. The nine screened domains are pulmonary, infectious, cardiovascular, neurological, renal, gastrointestinal, wound, pain, and hematological morbidities [17]. Since the POMS does not grade the severity of each complication, we additionally grade each complication using the Clavien and Dindo scale (Table 1) [18]. The primary endpoint is the frequency of patients with at least one clinically relevant postoperative complication, graded as Clavien-Dindo Score ≥ 2, on at least one postoperative day until hospital discharge.

**Table 1:** Definition of the Clavien-Dindo Score [18]

Grades	Definition
**Grade I**	Any deviation from the normal postoperative course without the need for pharmacological treatment or surgical, endoscopic, and radiological interventions.
	Allowed therapeutic regimens are drugs as antiemetics, antipyretics, analgesics, diuretics, and electrolytes and physiotherapy. This grade also includes wound infections opened at the bedside.
**Grade II**	Requiring pharmacological treatment with drugs other than such allowed for grade I complications. Blood transfusions and total parenteral nutrition are also included.
**Grade III**	Requiring surgical, endoscopic, or radiological intervention.
**Grade III-a**	Intervention not under general anesthesia.
**Grade III-b**	Intervention under general anesthesia.
**Grade IV**	Life-threatening complication (including CNS complications) requiring IC/ICU management.
**Grade IV-a**	Single organ dysfunction (including dialysis).
**Grade IV-b**	Multi-organ dysfunction.
**Grade V**	Death of a patient.

Secondary outcome variables are the subjective quality of recovery, in-hospital and 180-day mortality, length of stay in the PACU, intensive care unit and hospital, incidence of complications and the respective surrogate variables in the nine POMS domains (e.g., acute kidney injury, pulmonary congestion, myocardial injury after noncardiac surgery, perioperative catecholamine requirements, hypotension, infusion, and transfusion), and the efficiency of the albumin substitution.

The quality of recovery is assessed using the Quality of Recovery-9 (QoR-9) Questionnaire preoperatively and postoperatively on days 1, 3, and 180. The QoR-9 Questionnaire is a nine-item scale to determine subjective recovery after surgery (Table 2) in its German version [22, 23]. Total QoR-9 scores range from 0 to 18, with higher scores indicating better recovery.

**Table 2:** Quality of Recovery (QoR-9) Questionnaire. Each question can be quoted with 0, 1, or 2 according to the level of agreement. The total score is the sum of all responses. As the last three questions are asked inversely, the scoring scheme is to be inverted

QoR-9 questions	
Had a feeling of general well-being.	
Had support from others (especially doctors and nurses).	
Been able to understand instructions and advice. Not being confused.	
Been able to look after personal toilet and hygiene unaided.	
Been able to pass urine and having no trouble with bowel function.	
Been able to breathe easily.	
Been free from headache, backache, or muscle pains.	
Been free from nausea, dry retching, or vomiting.	
Been free from experiencing severe pain or constant moderate pain.	

Albumin in serum is measured photometrically on a Cobas 8000 c702 analyzer using the original reagent kit (Roche Diagnostics, Mannheim, Germany). High-sensitive cardiac troponin T in serum is measured using a sandwich electrochemiluminescent immunoassay on a Cobas 8000 e602 analyzer and original reagent kits (Roche Diagnostics, Mannheim, Germany). Immature platelets are measured using a fully automated analyzer (Sysmex XE-5000, Sysmex, Kobe, Japan). The device uses two fluorescent dyes containing polymethine and oxazine, which penetrate the cell membrane staining the RNA. Stained cells are then passed through a semiconductor laser beam, allowing differentiation between immature and mature platelets by plotting forward scatter light (measuring cell volume) against fluorescence intensity (representing RNA content) [24].

### Participant timeline

Patients are screened for eligibility, informed about the study, and asked for their consent during the preoperative anesthesia evaluation by any qualified anesthesiologist on duty. After arrival in the operation theater, albumin assessment starts as the second step of the screening process. For this purpose, albumin concentration is determined before the induction of anesthesia and at least every 3 h until admission to PACU. If the albumin concentration does not drop below 30 g/l, the patient will not be recruited. If the albumin concentration drops below 30 g/l, the patient is to be randomly allocated to the intervention or the control treatment. The randomized patients’ albumin concentration is to be assessed again every 3 h, as well as after each albumin infusion, until the intervention period ends with discharge from the PACU. Postoperative visits are to be performed on days 1, 3, 5, 8, and 15, unless the patient is previously discharged from the hospital. The patient will be contacted via phone 6 months after surgery.

### Allocation

The randomization list was generated by SJS using a 1:1 ratio and random blocks of 4, 8, or 16 using www.randomization.com. There was no stratification. The randomization list was generated using the same technique after the interim analysis. The list was transferred to an external contractor, which established a password-restricted internet platform on a university server in a validated process to ensure the correct order. Each member of the randomization team (BJ, KF, MB, SJS, or SS) holds an individualized login enabling randomization. The randomization team is available 24/7 via the study telephone. If a patient is to be randomized, the treating anesthesiologist calls the study telephone and provides the albumin level (to ensure that randomization is possible) and a 7-digit case identification number of the hospital record system. The randomization team checks if the signed informed consent is documented and performs the randomization in the online system. The allocation “intervention” or “standard care” is then reported back to the anesthesiologist via the phone. The anesthesiologist then documents the randomization number in the electronic anesthesia report. In case of technical issues with the online randomization, closed envelopes with printed randomization numbers are available in a locked safe at the intensive care unit of the department, accessible 24/7 through the head nurse of the ICU if necessary. The anesthesiologist can open the envelope and find the respective group assignment.

### Recruitment

The stepwise inclusion process is recognized a priori as a bottleneck for recruitment. Therefore, an emphasis was placed in defining a clear sequence of measures and responsibilities. First, we explicitly defined the population to be screened; the patients at surgical risk of losing albumin, specified as a list of high-risk surgical procedures or patients at risk for slow albumin synthesis, specified using the ASA risk score ≥ 3. Accordingly, we trained all anesthesiologists of the department in the correct use of the ASA risk score and the list of high-risk surgical procedures at least every 4 months. Second, we recruit patients of specified surgical departments operating in the same operation theater with 10 operation rooms. The attending anesthesiologists are trained to guarantee sampling of blood according to the given timelines, to ensure their appropriate labeling, to blind the surgical staff, and to prioritize albumin analysis in the department of clinical chemistry. Third, the anesthesiologists are quickly informed about the albumin concentration using telephone- and IT-based information pathways. Finally, the investigational product is prepared to be rapidly delivered by the transfusion department.

### Strategies to improve adherence to intervention protocols

We provide regular lectures for the participating departments. The randomization team can be contacted at any time to assist the anesthesiologists in charge with the requirements of the study protocol, in particular with the intervention. Furthermore, the study protocol is published in our in-house network, which is available on the monitor of the patient data management system. Finally, each anesthesiologist is equipped with a pocket card including major information about the study-related requirements.

### Criteria for discontinuing or modifying allocated interventions

Allergic reactions against the albumin preparation must be reported as a severe adverse event. If this is suspected, the trial medication is to be stopped immediately. Protocol violations, such as infusion of fresh frozen plasma or albumin preparations outside the study intervention, do not lead to termination of participation in the study.

### Sample size

The frequency of complications graded as Clavien-Dindo Score > 2 in surgical patients with preoperative hypalbuminemia has been reported as 52%, compared to 23% in patients with normal albumin concentration, according to an observational study in patients with ovarian cancer [3]. We assumed a clinically relevant treatment effect if albumin substitution could halve the absolute risk difference between patients with hypalbuminemia and normal albumin, i.e., an absolute risk reduction of 14% or a relative risk reduction of approximately 25%. Since a general surgical population was likely to have a different risk of complications related to hypalbuminemia, an interim analysis was planned after 100 patients, so as to better estimate the risk of complications in the study population and recalculate the sample size using a 25% relative risk reduction.

After inclusion of 100 patients, an independent statistician compared the two treatment groups for the incidence of any postoperative complications graded as Clavien-Dindo ≥2 and > 2, using a two-sided *χ*^2^ test for independent samples. Had the difference between groups reached a significance level of 0.002, the study would have been terminated. Since the incidences of complications graded as Clavien-Dindo ≥ 2 after 100 patients were 82% vs. 84% (*p* = 0.79) and those scored Clavien-Dindo > 2 were 34% vs. 36% (*p* = 0.83), the study must be continued. Hence, the 95% confidence interval of the relative risk for complications graded Clavien-Dindo ≥ 2 includes the postulated 25% relative risk reduction (0.87 [0.37–2.03]), the interim analysis also failed to show a missing treatment effect, which would also terminate the study. Based on incidences of the interim analysis, a significance level of 0.048, and a power of 80%, a sample size of 4408 patients would be required to prove significance. In fact, the study was limited to 300 patients per group, according to the prerequisite given by the responsible authority, the Paul-Ehrlich-Institut. Based on the limited number of 2 × 300 patients, the frequencies of complication of 82% and 84% and a significance level of 0.048, the power would be 50%.

### Data collection methods

Database management is delegated to the Munich Study Center, Munich, Germany (MSZ). First, data management and data verification plans were generated, followed by the creation of a validated study database using the platform EDC-System Macro (Elsevier, London, UK). Data electronically available in the anesthesia protocol is transferred into the database using a validated process. Screening, medical history, and PACU data is entered manually into the database by unblinded study staff. Blinded outcome data is entered in specified study iPads (Apple Inc., Cupertino, CA, USA) using FileMaker 16 Go (Claris International Inc. c/o Apple, Santa Clara, CA, USA) with a validated transfer process into the study database.

There is a prespecified monitor plan in place, including five on-site visits and remote monitoring every 6 weeks. Execution is delegated to the MSZ. On-site monitoring consists of source data verification and source data review, while data review will be performed remotely. The frequency of visits can be adapted in case of significant recruitment delays or other issues arise.

All study database users receive required training applicable to their role in the conduction of the trial. The completion of this training is tracked and attested by an instructor, who also review and verify the appropriate conduction of the trial and adherence to data accuracy and security goals.

### Data management

All data entered into the database is reviewed. Data cleaning is planned in three rounds to resolve discrepancies, search for missing data, and declare them as not available should no solutions be found. Furthermore, protocol violations are identified and classified. Withdrawal of the consent before anesthesia, i.e., before recruitment and intervention, is reported in the CONSORT diagram only. Withdrawal of the consent after intervention is also reported, but will not affect the study related analysis of the already collected data, as well as all safety-related events even after withdrawal. Loss of contact information may result in loss of follow-up information, which can be reduced through inquiries to the respective municipal registration offices.

The database will be locked and exported for statistical analysis. At this stage, permission for access to the database will be revoked for all investigators, and the database will be archived.

Missing information on the outcome variables, irrespective of the reason, results in the exclusion of a patient. If more than 5% of patients are excluded from the primary analysis, the study aim cannot be reached.

### Statistical methods

The statistical analysis plan is described in the study protocol. This version and its amendments are published at http://www.superadd.de, and any further updates will be made available there. After closing of the database, no changes to the analysis plan will be allowed.

Scaled variables will be presented, depending on their distribution, as mean and standard deviation or median and interquartile range. Categorical variables will be represented as numbers and frequencies.

Statistical analyses will be performed for all given outcomes, irrespectively of any protocol violation (intension-to-treat analysis). In case of major protocol violations or discontinuation due to treatment-specific complications, an exploratory patient-per-protocol analysis will be performed, excluding the respective patients.

The primary endpoint is the incidence of any postoperative complications graded as Clavien-Dindo ≥ 2. The two treatment groups will be compared using a two-sided *χ*^2^ test for independent samples regarding the incidence of any postoperative complication. The results will be presented as absolute risk reduction based on the Mantel-Haenszel risk differences and its improved confidence interval estimate [25]. Sub-groups analyses and adjustments will be included for risk groups according to ASA status, preoperatively existing vs. intraoperatively acquired hypalbuminemia, and varying surgical interventions. Exploratory comparisons will be performed of each component of the Clavien-Dindo, as well as each domain of the POMS. Scaled surrogate variables of fluid therapy, such as creatinine concentration, vasopressor infusion rates, or need for supplementary oxygen during PACU, will be compared with either *t* tests or Mann-Whitney *U* tests, according to the distribution of the variable.

Statistical significance will be assumed at an alpha level of 0.05. Statistical analyses will be conducted using Statistical Package for the Social Sciences 26.0 (IBM SPSS Statistics, Chicago, IL, USA).

### Sub-studies

We expect that the albumin concentration of approximately 20% of the screened patients will drop below 30 g/l. We will also analyze the serum samples of the remaining 80% (when screening for albumin) and will evaluate their primary and secondary outcomes. These data will be collected prospectively to allow secondary and exploratory analyses. All collected serum samples will be analyzed for immature platelets and hypersensitive troponin T concentrations. Planned sub-studies will evaluate the predictive value of these biomarkers on postoperative complications [26, 27]. Furthermore, we will evaluate whether postoperative complications can be detected earlier using the QoR-9 instrument repetitively in this population [28].

## Discussion

SuperAdd is the first clinical trial to investigate the effect of a goal-directed albumin substitution in a surgical population. Although hypalbuminemia is associated with an increased risk of postoperative complications, the substitution of the deficit is controversial due to the large number of clinical conditions causing hypalbuminemia and the diversity of treatment protocols. For instance, albumin substitution in intensive care patients with low albumin concentrations could not improve outcomes, although more stable hemodynamic conditions were reported [10–12]. Likewise, prophylactic albumin substitution for 3 days in surgical patients could not reduce postoperative complications [15]. However, the study could not even prove any surrogate effect of the treatment. The daily substitution was 20 g albumin, which was so meager that the concentrations continued to drop in the 64 treated patients (22.5 ± 4.8 g/l) compared to the 63 control patients (21.9 ± 4.5 g/l), far from enough to show any outcome effects.

Haynes and colleagues analyzed 79 randomized clinical trials about albumin substitution in 2003 [29]. Their metanalyses included 9 studies between 1948 and 1997, in which hypalbuminemia indicated albumin substitution, whereas only one took place in a surgical setting. Nevertheless, the metanalysis reported a consistent dose-dependency and suggested a clinical benefit when the attained albumin concentration exceeded 30 g/l. Recently, the dose-dependency of the effect of albumin substitution was confirmed in cardiac surgical patients. Administration of albumin immediately prior to surgery, aiming to increase the albumin level above 40 g/l, was shown to reduce the risk of acute kidney injury after off-pump coronary artery bypass surgery [14].

The primary outcome for this trial will be the frequency of postoperative complications. We selected the Clavien-Dindo Score [18] in combination with the POMS [17], which has been announced by the COMPAC group as the best choice to measure organ failure and survival in the context of the initiative to define *Standardised Endpoints in Perioperative Medicine (StEP)* [30]. While the Clavien-Dindo Score allows grading of postoperative complications, the POMS systematically addresses which organ system out of nine relevant domains is affected, as well as at which time after surgery it first develops. The combination of both instruments will allow us to properly characterize the complications and to understand how long the treatment effect can be seen. Furthermore, exploratory analyses using each domain as an independent endpoint may allow for the characterization of benefits or harms of the intervention in each of the specified organ systems. For instance, fluid may reduce acute kidney injury while increasing the risk of lung edema.

A major limitation of the study design is the sample size calculation. The prevalence of postoperative complications is given, but not in combination with hypalbuminemia in a general surgical population. Therefore, we performed an interim analysis to better estimate the incidence of complications and to get a figure of potential treatment differences. After 100 patients, the differences were so small that approximately 9000 patients would be required for the study and an expected number-needed-to-treat of 50 [28–232]. The study cohort size to answer the research question was restricted a priori by the regulatory authority to a maximum of 600 patients. In fact, we agree that if there is no beneficial effect of albumin substitution in the cohort of 600 patients with a high risk of complications and given hypalbuminemia, the significant costs of the substitution do not justify further consideration.

We are screening two specific cohorts with an expected high incidence of perioperative hypalbuminemia, due to either an increased loss or a decreased synthesis of albumin. The specified surgical procedures have a transfusion risk > 20% in our hospital and patients with an ASA risk score ≥ 3 are known to produce albumin at a reduced rate [5]. With approximately 1200–1500 patients per year fulfilling the eligibility criteria at the operation units of the Klinikum rechts der Isar, we expect to enroll the 600 patients during a recruitment period of 3 years. Accordingly, approximately 4000 patients will be screened. The nonrestrictive selection guarantees that the patients without albumin screening will most likely not develop hypalbuminemia. Therefore, the high effort and workload promises generalizability of the study results.

### Trial status

The first patient was randomized on 25 June 2017. The interim analysis was completed on 13 November 2018. Recruitment will continue until the complete sample size is achieved, which is expected to be in June 2020.

## Supplementary information

**Additional file 1.** SPIRIT 2013 Checklist: Recommended items to address in a clinical trial protocol and related documents.

**Additional file 2.**

## Data Availability

The Department of Anesthesiology and Intensive Care of the School of Medicine, Technical University of Munich, will handle the data analysis. The study database, monitoring, and safety reporting are operated by the Munich Study Center (MSZ). Severe adverse events are to be immediately reported to MSZ. A yearly safety report is generated and distributed to the national authority (Paul-Ehrlich-Institute, Bonn, Germany). Datasets can be made available on reasonable request.
