# Supplementary material for: Substitution of perioperative albumin deficiency disorders (SuperAdd) in adults undergoing vascular, abdominal, trauma, or orthopedic surgery: protocol for a randomized controlled trial
Source: Trials. 2020 Aug 18;21:726. doi: 10.1186/s13063-020-04626-2 (PMC7433099; doi:10.1186/s13063-020-04626-2)
Supplement: Supplementary file 2 — Additional file 2. [file 13063_2020_4626_MOESM2_ESM.pdf]

Prüfstelle: **Technische Universität München**
Klinikum rechts der Isar
Klinik für Anästhesiologie und Intensivmedizin
Ismaningerstr. 22
81675 München
Telefon: 089-4140-4291

Prüfarzte: **Prof. Dr. Manfred Blobner, Prof. Dr. Bettina Jungwirth, Dr. Kristina Fuest, Dr. Sebastian Schmid, Dr. Stefan Schaller, Catherina Bubb**

EUDRACT-Nr.: **2016-001313-24**

Patienteninformation
Einfluss der Therapie einer perioperativen Hypoalbuminämie auf postoperative Komplikationen

(SUPERADD)

Sehr geehrte Patientin, sehr geehrter Patient,

wir möchten Sie fragen, ob Sie bereit sind, an der nachfolgend beschriebenen klinischen Prüfung (Studie) teilzunehmen.

Ihre Teilnahme an dieser klinischen Prüfung ist freiwillig. Sie werden in diese Prüfung also nur dann einbezogen, wenn Sie dazu schriftlich Ihre Einwilligung erklären. Sofern Sie nicht an der klinischen Prüfung teilnehmen oder später aus ihr ausscheiden möchten, erwachsen Ihnen daraus keine Nachteile.

Sie wurden bereits auf die geplante Studie angesprochen. Der nachfolgende Text soll Ihnen die Ziele und den Ablauf erläutern. Anschließend wird ein Prüfarzt das Aufklärungsgespräch mit Ihnen führen. Bitte zögern Sie nicht, alle Punkte anzusprechen, die Ihnen unklar sind. Sie werden danach ausreichend Bedenkzeit erhalten, um über Ihre Teilnahme zu entscheiden.

1. Warum wird diese Prüfung durchgeführt?

Bei jeder größeren Operation kann es entweder schon während der Operation oder später bei der Überwachung im Aufwachraum zu Eiweißmangelzuständen kommen. Diese sind entweder durch eine schwere Krankheit wie eine Krebserkrankung oder Mangelernährung verursacht oder entstehen durch die Operation selbst. Es wird diskutiert ob dieser Eiweißmangel Komplikationen nach der Operation begünstigen kann und damit die Erholungszeit nach einer großen Operation verlängern kann.
Zum gegenwärtigen Zeitpunkt können noch keine Empfehlungen ausgesprochen werden, wie diese Eiweißmangelzustände sicher zu vermeiden oder zu behandeln sind. Es wird aber vermutet, dass eine gezielte Gabe von Eiweißpräparaten während der Narkose von entscheidender Bedeutung ist, wenn man Eiweißmangel verhindern will.
Das wichtigste Eiweiß im Körper ist Albumin. Dieses gibt es als Infusionslösung (Humanalbumin). In dieser Infusion ist eine große Menge von Eiweiß enthalten, die den Mangel im Körper schnell wieder ausgleichen kann.
Mit dieser Untersuchung wollen wir zeigen, dass bei Patienten die Behandlung eines Eiweißmangels durch Albumin die durch die Operation verursachten Komplikationen verhindern kann.

2. Erhalte ich das Prüfpräparat auf jeden Fall?

Im Rahmen dieser klinischen Prüfung wird die Behandlung eines Eiweißmangels mit der Nicht-Behandlung verglichen. Insgesamt sollen 600 Patienten aufgenommen werden, d.h. 300 Patienten werden die eiweißhaltige Infusionslösung erhalten und 300 werden diese nicht erhalten.
Zur objektiven Gewinnung von Studiendaten ist es notwendig, dass weder Sie noch Ihr Prüfarzt wissen, ob Sie das Medikament erhalten haben. Sollte es aus Sicherheitsgründen notwendig sein, kann unverzüglich festgestellt werden, ob Sie in der Behandlungsgruppe gewesen sind.

3. Wie ist der Ablauf der Studie und was muss ich bei Teilnahme beachten?

Die Studie beginnt mit der Vorbesprechung, in der Sie über die Teilnahme, den Ablauf und die möglichen Risiken dieser Untersuchung aufgeklärt werden. Hier bewerten wir anhand Ihrer Vorerkrankungen und der Schwere der Operation, der Sie sich unterziehen müssen, ob das Risiko einen Eiweißmangel zu bekommen, für Sie sehr wahrscheinlich ist. Sollte dies so sein, wird vor Beginn der Narkose durch eine Blutabnahme Ihr persönlicher Eiweißwert (Albumin) bestimmt. Diese Blutabnahme erfolgt über die Infusionsnadel, die wir Ihnen ohnehin für die Narkose legen müssen, es ist also keine zusätzliche Punktion einer Vene nötig. Besteht bei Ihnen zu irgendeinem Zeitpunkt während der Operation ein Eiweißmangel, werden Sie zufällig einer von zwei Gruppen zugeteilt. In der einen Gruppe erhalten sie die eiweißhaltige Infusionslösung in der andern nicht. Durch die Studie wird ihr Aufenthalt im Krankenhaus nicht verlängert. Ihre Studienteilnahme endet mit dem Verlassen des Aufwachraumes.

4. Welchen persönlichen Nutzen habe ich von der Teilnahme an der Studie?

Sie haben einen persönlichen individuellen Nutzen aus der Teilnahme an der Studie. Zur Einhaltung des Studienprotokolls ist zeitweise ein zusätzlicher Anästhesist/Narkosearzt während der Narkose anwesend. Dies bedeutet eine zusätzliche Sicherheit für sie.

5. Welche Risiken sind mit der Teilnahme an der Studie verbunden?

Die eiweißhaltige Infusionslösung ist ein Medikament, dass es schon seit langem in Deutschland gibt und mit dem umfassende Erfahrungen bestehen. Zurzeit besteht kein Hinweis darauf, dass diese Flüssigkeit mit anderen Medikamenten, die Sie vielleicht einnehmen müssen, Wechselwirkungen eingeht. Weiterhin bestehen keine Nebenwirkungen, die Ihnen oder Ihren Organen körperlichen Schaden zufügen können. In seltenen Fällen ist eine allergische Reaktion beschrieben, die – wie auch bei allen anderen Medikamenten oder Substanzen – in unterschiedlichen Schweregraden auftreten kann. Es besteht die Möglichkeit einer Hautrötung und Juckreiz als Zeichen einer leichten allergischen Reaktion sowie in Einzelfällen das Risiko einer schweren sogenannten Anaphylaxie. In diesem Fall bekommen Sie von Ihrem betreuenden Narkosearzt unmittelbar Medikamente gegen diese allergische Reaktion verabreicht.
Es ergeben sich für Sie bei der Teilnahme an der Studie keine weiteren Risiken im Vergleich zu anderen Patienten mit einem ähnlichen operativen Eingriff. Die für die Narkose verwendeten Medikamente und Katheter werden in unserer Klinik täglich im Routinebetrieb eingesetzt.
Durch die Studie ergibt sich lediglich eine zusätzliche Blutabnahme zu Beginn der Narkose über die liegende Infusionsnadel. Dafür ist nur eine kleine Blutmenge nötig, die mit keinen weiteren Risiken verbunden ist. Die weiteren Laborkontrollen, die wir im Rahmen dieser Studie untersuchen, werden routinemäßig abgenommen, genau wie bei Patienten, die nicht an dieser Studie teilnehmen.

Bitte teilen Sie den Mitarbeitern der Prüfstelle *alle* Beschwerden, Erkrankungen oder Verletzungen mit, die im Verlauf der klinischen Prüfung auftreten. Falls diese schwerwiegend sind, teilen Sie den Mitarbeitern der Prüfstelle diese bitte umgehend mit, ggf. telefonisch.

6. Welche anderen Behandlungsmöglichkeiten gibt es außerhalb der Studie?

Falls Sie an extremen Untergewicht leiden, ist zu überlegen, ob Sie vor der Operation bereits eiweißhaltige Getränke zu sich nehmen.

7. Wer darf an dieser klinischen Prüfung nicht teilnehmen?

An dieser klinischen Prüfung dürfen Sie nicht teilnehmen, wenn Sie gleichzeitig an anderen klinischen Prüfungen oder anderen klinischen Forschungsprojekten teilnehmen oder vor kurzem teilgenommen haben.
Schwangere und stillende **Frauen** dürfen an dieser klinischen Prüfung **nicht teilnehmen**. Zu Beginn der klinischen Prüfung müssen sich deshalb alle Frauen, die eine Schwangerschaft nicht sicher ausschließen können, einem Schwangerschaftstest unterziehen. Davon ausgenommen sind Frauen nach den Wechseljahren oder solche, die operativ sterilisiert wurden. Durch einen Schwangerschaftstest kann jedoch eine Schwangerschaft erst einige Tage nach der Empfängnis verlässlich nachgewiesen werden.

8. Entstehen für mich Kosten durch die Teilnahme an der klinischen Prüfung? Erhalte ich eine Aufwandsentschädigung?

Durch Ihre Teilnahme an dieser klinischen Prüfung entstehen für Sie keine zusätzlichen Kosten.
Für Ihre Teilnahme an dieser klinischen Prüfung erhalten Sie KEINE Aufwandsentschädigung.

9. Werden mir neue Erkenntnisse während der klinischen Prüfung mitgeteilt?

Sie werden über neue Erkenntnisse, die in Bezug auf diese klinische Prüfung bekannt werden und die für Ihre Bereitschaft zur weiteren Teilnahme wesentlich sein können, informiert. Auf dieser Basis können Sie dann Ihre Entscheidung zur weiteren Teilnahme an dieser klinischen Prüfung überdenken.

10. Wer entscheidet, ob ich aus der klinischen Prüfung ausscheide?

Sie können jederzeit, auch ohne Angabe von Gründen, Ihre Teilnahme beenden, ohne dass Ihnen dadurch irgendwelche Nachteile bei Ihrer medizinischen Behandlung entstehen.
Unter gewissen Umständen ist es aber auch möglich, dass der Prüfarzt oder der Sponsor entscheidet, Ihre Teilnahme an der klinischen Prüfung vorzeitig zu beenden, ohne dass Sie auf die Entscheidung Einfluss haben. Die Gründe hierfür können z. B. sein:

- Ihre weitere Teilnahme an der klinischen Prüfung ist ärztlich nicht mehr vertretbar;
- es wird die gesamte klinische Prüfung abgebrochen.

Der Prüfarzt wird mit Ihnen besprechen, wie und wo Ihre weitere Behandlung stattfindet.

11. Was geschieht mit meinen Daten?

In dieser Studie ist die Medizinische Fakultät der Technischen Universität München vertreten durch den Dekan (Ismaningerstrasse 22, 81675 München, Tel: 089/4140-4022, Email: dekanat.medizin@tum.de) für die Datenverarbeitung der Teilnehmer am Klinikum rechts der Isar verantwortlich.
Zu den verwendeten Daten gehören personenidentifizierende Daten wie Name, Anschrift und sensible personenbezogene Gesundheitsdaten. Alle unmittelbar Ihre Person identifizierenden Daten (z.B. Name, Geburtsdatum und Anschrift) werden durch einen Identifizierungscode ersetzt (pseudonymisiert) und nicht weitergegeben. Dies schließt eine Identifizierung Ihrer Person durch Unbefugte weitgehend aus.
Ihre Daten werden am Klinikum rechts der Isar, der Technischen Universität München, dem Leibnitz Rechenzentrum und Münchner Studienzentrum gespeichert. Zu organisatorischen Zwecken können Ihre Kontaktdaten (ohne Gesundheitsdaten) temporär auch durch berechtigte Mitarbeiter in analoger Form oder mittels verschlüsselter End-zu-End Kommunikation verwendet sowie auf Endgeräten gespeichert werden. Eine anderweitige Weitergabe von Daten ist lediglich in anonymisierter Form (d.h. ohne jegliche personenbezogenen Daten und ohne Identifizierungscode) zulässig. Eine Löschung Ihrer Daten erfolgt gemäß der gesetzlichen Löschfristen.
Die Einwilligung zur Verarbeitung Ihrer Daten ist freiwillig, Sie können jederzeit die Einwilligung ohne Angabe von Gründen und ohne Nachteile für Sie widerrufen.
Sie haben das Recht, Auskunft über die Sie betreffenden Daten zu erhalten, auch in Form einer unentgeltlichen Kopie. Darüber hinaus können Sie die Berichtigung oder Löschung Ihrer Daten verlangen, solange diesem Anliegen nicht andere Vorschriften entgegenstehen. Wenden Sie sich in diesen Fällen an das Studienteam (Tel: 089/4140-5761).

Im Falle einer Beschwerde wenden Sie sich an

> Behördlicher Datenschutzbeauftragter, Klinikum rechts der Isar der Technischen Universität München, Ismaninger Str. 22, 81675 München
> E-Mail: datenschutz@mri.tum.de

oder an

> Bayerischer Landesbeauftragten für den Datenschutz, Postanschrift: Postfach 22 12 19, 80502 München, Hausanschrift: Wagmüllerstr. 1, 80538 München
> E-Mail: poststelle@datenschutz-bayern.de.

Einzelheiten, insbesondere zur Möglichkeit eines Widerrufs, entnehmen Sie bitte der Einwilligungserklärung, die im Anschluss an diese Patienteninformation abgedruckt ist.

12. Bevollmächtigung Abfrage Personenstandsregister

Wir ersuchen Sie uns ebenfalls zu ermächtigen, Auskunft bei den Personenstandsregistern einzuholen. Diese Erlaubnis nutzen wir nur, wenn wir sie nach 6 Monaten telefonisch nicht erreichen, um ihr Langzeitergebnis zu erfragen.

13. An wen wende ich mich bei weiteren Fragen?

Beratungsgespräche an der Prüfstelle

Sie haben stets die Gelegenheit zu weiteren Beratungsgesprächen mit dem auf Seite 1 genannten oder einem anderen Prüfarzt.

089/4140 5761

Kontaktstelle

Es existiert außerdem eine Kontaktstelle bei der zuständigen Bundesoberbehörde. Teilnehmer an klinischen Prüfungen, ihre gesetzlichen Vertreter oder Bevollmächtigten können sich an diese Kontaktstelle wenden:

Paul-Ehrlich-Institut
Bundesinstitut für Impfstoffe und biomedizinische Arzneimittel
Referat Klinische Prüfung
Paul-Ehrlich-Str. 51-59
63225 Langen

Telefon: 06103/77 1810 Fax: 06103/77 1277
e-mail: klinpruefung@pei.de

Prüfstelle: **Technische Universität München**
Klinikum rechts der Isar
Klinik für Anästhesiologie und Intensivmedizin
Ismaningerstrasse 22
81675 München
Telefon: 089-4140-4291
Prüfarzte: **Prof. Dr. Manfred Blobner, Prof. Dr. Bettina Jungwirth, Dr. Kristina Fuest, Dr. Sebastian Schmid, Dr. Stefan Schaller, Catherina Bubb**

EUDRACT-Nr.: **2016-001313-24**

Einfluss der Therapie einer perioperativen Hypoalbuminämie auf postoperative Komplikationen

(SUPERADD)

Einwilligungserklärung

Patientenetikett

Ich bin in einem persönlichen Gespräch durch den Prüfarzt

....
Name der Ärztin/des Arztes

ausführlich und verständlich über das Prüfmedikament und die Vergleichstherapie sowie über Wesen, Bedeutung, Risiken und Tragweite der klinischen Prüfung aufgeklärt worden. Ich habe darüber hinaus den Text der Patienteninformation sowie die hier nachfolgend abgedruckte Datenschutzerklärung gelesen und verstanden. Ich hatte die Gelegenheit, mit dem Prüfarzt über die Durchführung der klinischen Prüfung zu sprechen. Alle meine Fragen wurden zufrieden stellend beantwortet.

Möglichkeit zur Dokumentation zusätzlicher Fragen seitens des Patienten oder sonstiger Aspekte des Aufklärungsgesprächs:

____

____

____

____

Ich hatte ausreichend Zeit, mich zu entscheiden.

Mir ist bekannt, dass ich jederzeit und ohne Angabe von Gründen meine Einwilligung zur Teilnahme an der Prüfung zurückziehen kann (mündlich oder schriftlich), ohne dass mir daraus Nachteile für meine medizinische Behandlung entstehen.

Datenschutz:

Mir ist bekannt, dass bei dieser klinischen Prüfung personenbezogene Daten, insbesondere medizinische Befunde über mich erhoben, gespeichert und ausgewertet werden sollen. Die Verwendung der Angaben über meine Gesundheit erfolgt nach gesetzlichen Bestimmungen und setzt vor der Teilnahme an der klinischen Prüfung folgende freiwillig abgegebene Einwilligungserklärung voraus, das heißt ohne die nachfolgende Einwilligung kann ich nicht an der klinischen Prüfung teilnehmen.

1. Ich erkläre mich damit einverstanden, dass im Rahmen dieser klinischen Prüfung personenbezogene Daten, insbesondere Angaben über meine Gesundheit, über mich erhoben und in Papierform sowie auf elektronischen Datenträgern am Klinikum rechts der Isar aufgezeichnet werden. Soweit erforderlich, dürfen die erhobenen Daten pseudonymisiert (verschlüsselt) weitergegeben werden:
   a) an die Technische Universität München, den Sponsor oder eine von diesem beauftragte Stelle zum Zwecke der wissenschaftlichen Auswertung,
   b) im Falle unerwünschter Ereignisse: an die Technische Universität München, den Sponsor, an die jeweils zuständige Ethik-Kommission und die zuständige Bundesoberbehörde (Bundesinstitut für Arzneimittel und Medizinprodukte), sowie von dieser an die Europäische Datenbank.

2. Außerdem erkläre ich mich damit einverstanden, dass autorisierte und zur Verschwiegenheit verpflichtete Beauftragte des Sponsors sowie die zuständigen Überwachungsbehörden in meine beim Prüfarzt vorhandenen personenbezogenen Daten, insbesondere meine Gesundheitsdaten, Einsicht nehmen, soweit dies für die Überprüfung der ordnungsgemäßen Durchführung der Studie notwendig ist. Für diese Maßnahme entbinde ich den Prüfarzt von der ärztlichen Schweigepflicht.

3. Die Einwilligung zur Erhebung und Verarbeitung meiner personenbezogenen Daten, insbesondere der Angaben über meine Gesundheit, ist unwiderruflich. Ich bin bereits darüber aufgeklärt worden, dass ich jederzeit die Teilnahme an der klinischen Prüfung beenden kann. Im Fall eines solchen Widerrufs meiner Einwilligung, an der Studie teilzunehmen, erkläre ich mich damit einverstanden, dass die bis zu diesem Zeitpunkt gespeicherten Daten weiterhin verwendet werden dürfen, soweit dies erforderlich ist, um
   a) Wirkungen des zu prüfenden Arzneimittels festzustellen,
   b) sicherzustellen, dass meine schutzwürdigen Interessen nicht beeinträchtigt werden,
   c) der Pflicht zur Vorlage vollständiger Prüfungsunterlagen zu genügen.

4. Ich erkläre mich damit einverstanden, dass meine Daten nach Beendigung oder Abbruch der Prüfung mindestens zehn Jahre aufbewahrt werden, wie es die Vorschriften über die klinische Prüfung von Arzneimitteln bestimmen. Danach werden meine personenbezogenen Daten gelöscht, soweit nicht gesetzliche, satzungsmäßige oder vertragliche Aufbewahrungsfristen entgegenstehen.

5. Ich bin über folgende gesetzliche Regelung informiert: Falls ich meine Einwilligung, an der Studie teilzunehmen, widerrufe, müssen alle Stellen, die meine personenbezogenen Daten, insbesondere Gesundheitsdaten, gespeichert haben, unverzüglich prüfen, inwieweit die gespeicherten Daten für die in Nr. 3 a) bis c) genannten Zwecke noch erforderlich sind. Nicht mehr benötigte Daten sind unverzüglich zu löschen.

6. Ich bin damit einverstanden, dass die für die Studie notwendigen Daten pseudonymisiert am Klinikum rechts der Isar, dem Münchner Studienzentrum, der Technischen Universität München sowie dem Leibnitz Rechenzentrum gespeichert und ausgewertet werden. Mir ist bewusst, dass eine temporäre Verwendung und Speicherung der Kontaktdaten in analoger Form, mittels verschlüsselter End-zu-End Kommunikation oder auf Endgeräten von Studienmitarbeitern erfolgen kann.

7. Ich bin damit einverstanden, dass meine pseudonymisierten Daten für weitere wissenschaftliche Auswertungen von den Prüfärzten verwendet werden dürfen.

Bevollmächtigung Abfrage Personenstandsregister

Ich bevollmächtige mit meiner Unterschrift die Prüfärzte der SuperAdd Studie Abfragen an die Personenstandsregister zu meiner Person zu stellen.

☐ Zustimmung ☐ Ablehnung

Ich erkläre mich bereit,
an der oben genannten klinischen Prüfung
freiwillig teilzunehmen.

Ein Exemplar der Patienten-Information und -Einwilligung habe ich erhalten. Ein Exemplar verbleibt im Prüfzentrum.

Patientenetikett

....
Datum Unterschrift des **Patienten**

Ich habe das Aufklärungsgespräch geführt und die Einwilligung des Patienten eingeholt.

....
Name des Prüfarztes/der Prüfärztin in Druckbuchstaben

....
Datum Unterschrift des aufklärenden **Prüfarztes/der Prüfärztin**
